# PDMS SlipChip: Optimizing Sealing, Slipping, and Biocompatibility Using Low-Viscosity Silicone Oils

**DOI:** 10.3390/mi16050525

**Published:** 2025-04-29

**Authors:** Rafia Inaam, Marcela F. Bolontrade, Shunya Okamoto, Takayuki Shibata, Tuhin Subhra Santra, Moeto Nagai

**Affiliations:** 1 Department of Mechanical Engineering, Toyohashi University of Technology, Toyohashi 441-8580, Japan; inaam.rafia.ir@tut.jp (R.I.); okamoto@me.tut.ac.jp (S.O.); shibata@me.tut.ac.jp (T.S.); 2 Institute of Translational Medicine and Biomedical Engineering (IMTIB), CONICET, Italian Hospital University—Italian Hospital of Buenos Aires, Buenos Aires C1199ACL, Argentina; marcela.bolontrade@hospitalitaliano.org.ar; 3 Department of Engineering Design, Indian Institute of Technology Madras, Chennai 600036, India; tuhin@zmail.iitm.ac.in; 4 The Institute for Research on Next-Generation Semiconductor and Sensing Science (IRES^2^), Toyohashi University of Technology, Toyohashi 441-8580, Japan

**Keywords:** PDMS SlipChip, sealing performance, curing temperature, silicone oil lubrication, concentration gradients, biocompatibility

## Abstract

The Polydimethylsiloxane (PDMS) SlipChip is a microfluidic platform enabling fluid manipulation without pumps or valves, simplifying operation and reducing reagent use. High-viscosity silicone oils (e.g., 5000–10,000 cSt) improve sealing but frequently block microfluidic channels, reducing usability. In contrast, low-viscosity oils (50–100 cSt) reduce blockages but may compromise sealing. This study addresses these challenges by optimizing the viscosity of silicone oil and the curing conditions of PDMS. Low-viscosity silicone oil (50 cSt) was identified as optimal, ensuring smooth slipping and reliable sealing without blockages. Curing conditions were also adjusted to balance adhesion and stiffness as follows: lower temperatures (50–60 °C) enhanced van der Waals adhesion, while higher temperatures (80 °C) increased stiffness. A mixed curing approach (80 °C for the top layer and 60 °C for the bottom layer) further improved performance. Biocompatibility testing using human osteosarcoma cells demonstrated minimal cytotoxicity with 50 cSt oil, supporting cell viability (95%) comparable to traditional multiwell plates. These findings provide practical guidelines for fabricating reliable and biocompatible SlipChips.

## 1. Introduction

Investigating the intricacies of chemistry and biology often requires careful surveys using minimal reagents [1,2,3]. Conventional microfluidic devices require pumps and valves for biological and chemical assays [4,5,6].To remove the external components, centrifugal microdevices [7,8] or paper-based microfluidic devices [9] are used. The SlipChip is a simple microfluidic device consisting of two aligned layers with flow channels and microwells, enabling researchers to perform operations efficiently [10,11,12,13]. The SlipChip operates solely based on a ‘slip action’ for fluid manipulation. This platform creates isolated reaction chambers with minimal sample consumption while maintaining high throughput through its ability to initiate, reroute, and isolate fluid flow by engaging or disengaging microfluidic channels. Glass SlipChips are commonly used due to their chemical resistance and precise fabrication [13]. However, their complex manufacturing process of etching with the use of hazardous chemicals and lack of gas permeability make them unsuitable for applications such as cell culture, where gas exchange is critical [14].

Polydimethylsiloxane (PDMS) SlipChips are a cost-effective and easily fabricated alternative [15]. Unlike glass SlipChips requiring harsh etching, PDMS versions are easily fabricated simply by molding. Precursors are mixed, cast onto a mold, and baked, precisely replicating features. This mild process enables rapid prototyping. Additionally, PDMS’s gas permeability and bio-inertness make it ideal for cell-based assays [16,17]. Despite these advantages, PDMS introduces challenges such as increased friction between layers. Silicone oil lubricants can reduce this friction and facilitate slipping, but excessive lubrication weakens adhesion, complicating controlled slip motion. High-viscosity silicone oils (5000 cSt [18] and 10,000 cSt [15,19,20]) have been used in previous studies to address slipping challenges by enhancing sealing performance. Additionally, the potential cytotoxicity associated with high-viscosity oils poses a challenge for cell-based applications, as substantial interaction between the oil and cells can compromise viability, rendering the device unsuitable for such work. On the other hand, low-viscosity oils are less likely to cause blockages but may reduce sealing performance. Therefore, it is crucial to explore strategies to balance these competing factors effectively.

A key factor influencing the performance of PDMS SlipChips is the cross-linking density of the PDMS material, which is determined by the curing temperature [21]. Lower curing temperatures enhance adhesion and reduce stiffness, while higher curing temperatures increase stiffness but diminish adhesion [22,23]. This behavior arises from tethered or free polymer chains on cured PDMS surfaces that interact via van der Waals forces during contact [24,25]. However, the effects of curing conditions on interlayer adhesion and their interplay with lubricant properties remain unclear. Further investigation is needed to clarify how curing conditions influence these interactions and optimize SlipChip performance.

The design of SlipChip enables the high-throughput generation of multiple drug concentrations and has been applied to biological assessments such as polymerase chain reaction (PCR) tests, bacterial detection, and drug development [26,27,28,29]. SlipChips show promise for drug uptake studies and disease biomarker measurements. While both glass and PDMS SlipChips have been used for antimicrobial testing, bacterial growth analysis, and antibiotic testing [11,12,13,15,27,29,30,31], research on mammalian cell growth in PDMS SlipChips remains limited. Although PDMS is an inert, non-toxic, and biocompatible material [32], some lubricants may exhibit cytotoxicity to mammalian cells, necessitating thorough biocompatibility testing.

This study investigates the use of low-viscosity silicone oils (50–100 cSt) as an alternative to high-viscosity oils, seeking to mitigate issues like channel blockages while maintaining effective sealing and slipping performance in PDMS SlipChip. The effects of PDMS curing temperatures on interlayer adhesion and stiffness were examined, identifying an optimal combination of 80 °C for the top layer and 60 °C for the bottom layer. Biocompatibility testing using human osteosarcoma cells demonstrated that 50 cSt silicone oil supports cell viability comparable to traditional multiwell plates while exhibiting minimal cytotoxicity. These findings offer practical guidelines for fabricating reliable and biocompatible SlipChips, demonstrating that low-viscosity lubricants effectively address performance trade-offs.

## 2. Materials and Methods

### 2.1. Design of PDMS SlipChip

**Figure 1:**
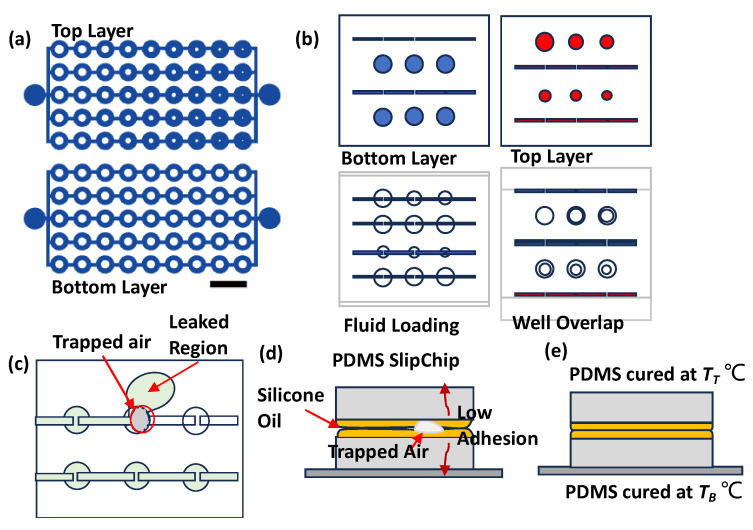
Design and characterization of PDMS SlipChip. (**a**) Test chip-like SlipChip with connected channels and obstructions for burst pressure measurement. Scale Bar: 4 mm. (**b**) SlipChip to develop gradient concentration. (**c**) Top view and (**d**) side view showing trapped air causing leakage in SlipChip. (**d**,**e**) Characterization with different curing temperatures.

The SlipChip design features two key configurations. Figure 1a illustrates a setup designed to evaluate the sealing performance of the PDMS interlayer. This configuration includes microwells (2 mm in diameter) connected by channels in both layers. The bottom layer incorporates obstructions of 0.9 mm, while the top layer features obstructions that gradually decrease in size from 0.9 mm to 0.12 mm. This design facilitates the detection of potential leak points efficiently.

Figure 1b represents a practical implementation for generating concentration gradients. The top layer contains six microwells with decreasing diameters of 3.0 mm, 2.6 mm, 2.2 mm, 2 mm, 1.8 mm, and 1.4 mm, with a uniform structure depth of 0.35 mm. This design facilitates fluid loading and diffusive mixing between layers. Figure 1b highlights the following design phases: configuration, loading, and mixing of two fluids.

The image also reveals some common operational challenges in Figure 1c,d. Air bubbles trapped between PDMS layers can block intended fluid paths, while insufficient sealing leads to fluid leakage in undesired directions. These observations highlight the critical role of sealing and air removal in SlipChip performance.

### 2.2. Fabrication of PDMS SlipChip

A SU-8 mold was fabricated on a silicon wafer using standard photolithography with SU-8 3050 negative photoresist [33]. The PDMS base and curing agent (Silpot 184 W/C by Dow Corning Toray Co., Ltd., Tokyo, Japan) were mixed at a ratio of 10:1 and poured onto the mold.

In all protocols, one layer was polymerized at 80 °C for one hour and fixed as a baseline. The other PDMS layers were subjected to five distinct temperature protocols to optimize their mechanical properties. Variations in the process involved steps applied to the other layer alone as follows: (i), (ii), (iii): The other layer was baked at 50 °C for two hours, 60 °C for two hours, or 80 °C for one hour, respectively. (iv), (v): The other layer underwent baking at 80 °C for 1 h and an additional thermal treatment at 100 °C or 120 °C for another hour, respectively. These protocols were designed to achieve specific mechanical properties, ensuring that the layer with additional treatment became slightly stiffer to facilitate handling during slipping. SlipChip performance was evaluated through leakage testing, pneumatic testing, and tensile testing across a range of curing temperatures as follows: 50 °C, 60 °C, 80 °C, 100 °C, and 120 °C. These temperatures were selected to balance efficiency and safety—lower temperatures were chosen to accelerate the curing process compared to room temperature, while higher temperatures were kept within a safe limit to ensure suitability for lab-scale fabrication and minimize the risk of accidents during student-led experiments.

Molds for gradient concentration SlipChips were fabricated using LCD VAT 3D printing (Sonic Mini 8K, Phrozen, Hsinchu, Taiwan). Poly (lactic acid) (PLA) frames were fabricated using an FDM 3D printer (Adventurer 4, FlashForge, Zhejiang, China) and mounted on top of the PDMS layer to enhance structural stability during slipping. The SlipChip itself was fabricated by soft lithography using a 10:1 ratio of the PDMS prepolymer.

### 2.3. Pneumatic Test for Separating Two Layers

A thin layer of silicone oil (120 mPa·s, 0.967 g/mL at 20 °C, #85409, Sigma Aldrich, St. Louis, MO, USA) was spin-coated onto the bottom layer at 1500 rpm to ensure uniform coverage. After alignment, the assembled SlipChip was placed under vacuum (–0.07 MPa) for 20 min to remove trapped air and enhance sealing between layers.

**Figure 2:**
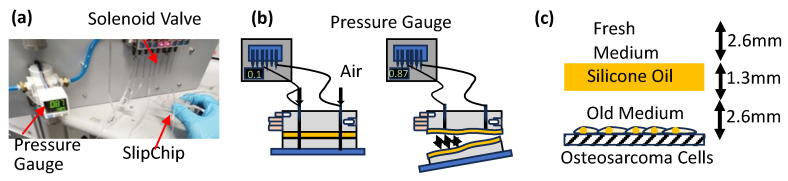
Pneumatic test on the SlipChip-mimicking device. (**a**) Apparatus used for the pneumatic test, showing the setup for applying air pressure to the SlipChip-mimicking device. (**b**) Schematic representation of the rupture mechanism caused by increasing air pressure applied to the inlet and outlet. (**c**) Biocompatibility testing of silicone oils with varying viscosities using a 96-well plate assay.

A pneumatic test was conducted to measure the adhesion between SlipChip layers and PDMS aging by determining the maximum pressure difference the SlipChip could withstand (Figure 2a). A 5 µM aqueous solution of Rhodamine 6G (R0039, Tokyo Chemical Industry Co., Ltd., Tokyo, Japan) [34] was prepared by dissolving the powder in water. A 0.01 M aqueous iodine solution was prepared by diluting a 0.5 M iodine solution (15-0610, Sigma-Aldrich, St. Louis, MO, USA) [35] with water. One of these solutions was selected and flowed through a test device that mimicked the actual SlipChip design. The device was then held in hand while pneumatic pressure was applied at both the inlet and outlet using a pneumatic pump. Gauge pressure was gradually increased from 0 kPa until the test device ruptured (Figure 2b), and the corresponding pressure readings were recorded as the rupture (burst) pressure. The SlipChip was then heated at 60 °C and experiments were repeated after 2 h each to determine the aging and adhesion strength of PDMS cured at different temperatures. Heating PDMS at 60 °C was chosen to accelerate PDMS aging and speed up the experimental process.

### 2.4. Tensile Testing of PDMS Cured at Different Temperatures

The mechanical properties of PDMS, specifically the stiffness (Young’s modulus) and elongation at break, are significantly affected by the curing temperature, which controls the material’s cross-linking density. Characterizing how these properties change with temperature is crucial for optimizing SlipChip performance by achieving the necessary balance between interlayer adhesion, facilitated by lower cross-linking, and structural integrity, provided by higher stiffness. To quantify these temperature-dependent mechanical characteristics, tensile tests were performed on PDMS samples. Molds for standard dumbbell-shaped specimens were designed using AutoCAD 3D 2021, fabricated via an LCD VAT 3D printer (Saturn3 Ultra 12K, ELEGOO, Shenzhen, China), and used to cast PDMS samples cured at 50 °C, 60 °C, 80 °C, 100 °C, and 120 °C.

The specimens measured 130 mm in overall length, with a 90 mm shoulder-to-shoulder distance, a 33 mm gauge length, and a 12.5 mm gauge width. The grip sections were 30 mm wide, and the overall thickness was 2 mm. Using a tensile testing machine (MCT-2150, A&D Co., Ltd., Tokyo, Japan), the prepared specimens were clamped in the grips and stretched until failure (breaking point). From the resulting stress–strain curve, the modulus of elasticity (Young’s modulus) was determined from the initial linear region, and the elongation at break was also recorded.

### 2.5. Characterization of Silicone Oil Properties: Leakage Testing and Layer Thickness Analysis

To analyze the silicone oil viscosity effects on SlipChip adhesion, we used devices fabricated with optimized curing conditions (top layer at 80 °C and bottom layer at 60 °C). Two ShinEtsu silicone oils with different viscosities were tested as follows: KF-96-50CS (50 cSt) and KF-96-100CS (100 cSt) (Shin-Etsu Chemical Co., Ltd., Tokyo, Japan). The oils were spin-coated onto the PDMS layers at the following four different speeds: 1000, 1500, 1800, and 2000 rpm. To evaluate sealing performance, we conducted leakage tests by connecting a micropipette to the SlipChip inlet and gradually increasing the iodine solution volume from 0 to 200 µL, with each loading step performed within 10 s.

To characterize the oil layer formation, deposition height measurements were performed using Z-stack imaging with an inverted fluorescence microscope (Nikon Eclipse Ti-E, Tokyo, Japan) and software NIS-Element AR (Nikon, 5.01.00, 64-bit, Tokyo, Japan). A total of 200 µL of each oil was placed at five different locations of the four different chips (center, top right, bottom right, top left, and bottom left) and spin-coated at 1000, 1500, 1800, and 2000 rpm. Each of the spin-coated chips was placed under the microscope and elevation levels were obtained by Z-stack imaging. The surface adjacent to the channel was focused and considered the top surface. The bottom surface was the reference plane (*Z* = 0) for channel depth. The elevation difference between the top and bottom surfaces determined the silicone oil deposition height.

### 2.6. Bio-Compatibility Test with Silicone Oil

We evaluated the biocompatibility of silicone oils using human osteosarcoma cells (SAOS-2, Tohoku University, Sendai, Japan) and their metastatic variant (LM-7, MD Anderson Cancer Center). Cells were seeded at a density of 100 cells/µL in Dulbecco’s Modified Eagle Medium (DMEM, High Glucose, Fujifilm Wako Co. Ltd., Osaka, Japan) in a flat bottom96-well plate (1-1601-06, Violamo, AS ONE Corporation, Osaka, Japan), with a total volume of 100 µL per well. The biocompatibility assessment focused on the diffusion of fresh culture medium and oxygen to the cells through the silicone oil layer, as well as the effects of direct contact and oil molecular weight. Cell viability was analyzed using a Calcein-AM and propidium iodide (PI) staining assay (Dojindo Laboratories, Kumamoto, Japan). Calcein-AM and PI solutions were diluted 1:10 in PBS before application. Images were captured using an inverted microscope (NIKON ECLIPSE Ti-E, Tokyo, Japan).

A layer of silicone oil was placed between the cancer cells and the cell culture medium to examine its effect on nutrient diffusion. Specifically, 50 µL of silicone oil (50 cSt or 100 cSt) was added above the culture medium 24 h after cell adhesion (Figure 2c). The silicone oil layer depth was maintained at 1.3 mm. Fresh medium was introduced from the top, followed by a 48 h incubation period. Live/dead cell assays were performed to assess cell viability, focusing on the diffusion of nutrients through the silicone oil layer. Additionally, the experimental setup allowed us to examine the impact of oil viscosity on nutrient diffusion.

### 2.7. Demonstration of the Diffusion Mechanism in the SlipChip Device

The mixing process in the SlipChip generates concentration gradients through a volume-based dilution system. The bottom layer contains uniform microwells (3.0 mm diameter and 2.5 mm^3^ volume), while the top layer features wells with decreasing diameters (3.0 mm to 1.4 mm), corresponding to volumes from 2.5 mm^3^ to 0.5 mm^3^. Both layers have a uniform depth of 0.35 mm.

The SlipChip design pairs microwells from the top and bottom layers to create controlled mixing chambers without introducing physical obstructions, minimizing the risk of bubble formation during fluid manipulation. As microwell sizes in the top layer decrease, the total volume in each well pair progressively reduces, allowing the formation of an intrinsic concentration gradient during mixing.

Using a SlipChip fabricated under optimized curing conditions (bottom layer at 60 °C, top layer at 80 °C), we generated concentration gradients with a 4 mM ethanol-based Rhodamine 6G solution. Fluorescence measurements were performed using an inverted microscope (Ti-E, Nikon, Tokyo, Japan) at 2× magnification. The concentration in each well was determined by the total mixing volume, with the initial well pair (5.0 mm^3^ total volume) yielding a concentration of 2.0 mM.

### 2.8. Demonstration of Cell Culture in SlipChip

A PDMS SlipChip was fabricated using a two-layer design as follows: the top layer was cured at 80 °C and the bottom layer at 60 °C, with a 50 cSt silicone oil layer applied between them to enable controlled slipping. SAOS-2 cells (Tohoku University, Sendai, Japan) were seeded at a density of 100 cells/µL in culture medium composed of Dulbecco’s Modified Eagle Medium (DMEM) supplemented with 10% fetal bovine serum (FBS, COSMO BIO Co., Ltd., Tokyo, Japan), 100 U/mL penicillin, and 100 µg/mL streptomycin (15070063, Gibco, Thermo Fisher Scientific, Waltham, MA, USA). The cells were maintained at 37 °C in a humidified atmosphere with 5% CO_2_.

After 24 h of incubation, cell viability was assessed using live/dead cell assays. Adhered cells were stained with Calcein-AM to label live cells and PI to label dead cells, followed by a 50 min incubation at 37 °C [36,37]. Calcein-AM is converted by intracellular esterase into green-fluorescent compounds in live cells, while PI binds to DNA in dead cells with damaged membranes, emitting red fluorescence. Fluorescence imaging was performed using an inverted microscope (NIKON ECLIPSE Ti-E, Tokyo, Japan). Comparison with SAOS-2 cells cultured in 96-well plates under the same conditions (with 50 cSt silicone oil) showed comparable cell viability and morphology between the two systems.

## 3. Results and Discussion

### 3.1. Pneumatic Test

**Figure 3:**
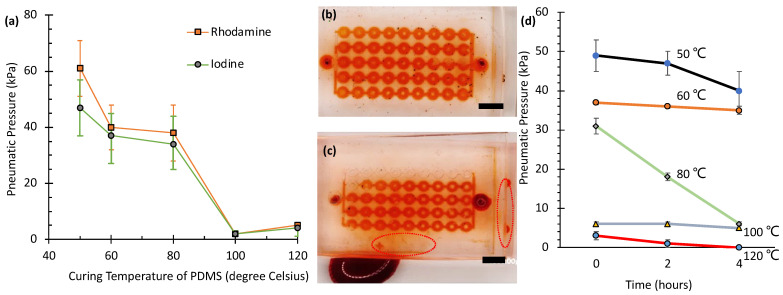
Adhesion measurements of PDMS SlipChip. (**a**) Pneumatic pressure measurements across different curing temperatures. (**b**,**c**) Leakage tests showing iodine solution flow in SlipChips using (**b**) 50 cSt and (**c**) 100 cSt silicone oil at 1000 rpm. Scale bars: 4 mm. Circles indicate leakage regions. (**d**) Effect of aging at 60 °C on the pneumatic pressure of PDMS samples initially cured for 2 h at different temperatures (50 °C, 60 °C, 80 °C, 100 °C, and 120 °C). Pressure was measured after 0, 2, and 4 h of aging at 60 °C. Results are presented as the mean ± standard deviation (*N* = 3).

Figure 3a shows the maximum pressures (rupture pressures) that a SlipChip, constructed with 120 mPa·s silicone oil applied by spin-coating at 1500 rpm, can withstand under various curing conditions. The sample, cured at 50 °C for two hours on one side, exhibited the highest rupture strength, requiring 61 ± 10 kPa (*N* = 21) for the rhodamine solution and 47 ± 10 kPa (*N* = 21) for the iodine solution. This superior performance is attributed to the reduced cross-linking density at lower curing temperatures, which increases the number of tethered or free polymer chains on the PDMS surfaces. These additional chains enhance van der Waals forces between layers, resulting in stronger adhesion and higher rupture resistance [22,23,24,25]. Similarly, the sample cured at 60 °C for two hours exhibited relatively high rupture pressures (40 ± 8 kPa, *N* = 16 for rhodamine; and 37 ± 10 kPa, *N* = 14 for iodine solutions). Although lower curing temperatures (e.g., 50 °C and 60 °C) enhance adhesion via increased van der Waals forces from a higher density of free polymer chains, they also produce softer PDMS layers with reduced structural integrity. With the combination of curing the bottom layer at 60 °C and the top layer at 80 °C, a balance between adhesion and stiffness is achieved.

In contrast, the sample with both sides cured at 80 °C for one hour (the standard curing condition) showed lower rupture pressures of 38 ± 10 kPa (*N* = 25) for rhodamine and 34 ± 9 kPa (*N* = 13) for iodine solutions, indicating that a higher cross-linking density reduces interlayer adhesion. Furthermore, samples subjected to additional curing at higher temperatures (100 °C and 120 °C) required the lowest pneumatic pressures to separate the layers—2 ± 1 kPa (*N* = 6) and 2 ± 1 kPa (*N* = 7) for rhodamine and iodine solutions at 100 °C, and 5 ± 1 kPa (*N* = 4) and 4 ± 1 kPa (*N* = 4) for rhodamine and iodine solutions at 120 °C, demonstrating that excessive cross-linking weakens adhesion.

Notably, no significant difference in rupture pressure was observed between the rhodamine and iodine solutions under any curing condition, as indicated by the overlapping error ranges. While both solutions exhibited varying rupture pressures under different curing temperatures, the differences between the two solutions at each temperature point fell within experimental uncertainty. This suggests that the adhesion strength is primarily governed by the PDMS cross-linking density rather than by the chemical nature of the test solutions.

Figure 3d presents the results of an aging study, evaluating the pneumatic sealing pressure of PDMS SlipChips over a 4 h period. All measurements were performed in triplicate (*N* = 3), with data presented as the mean ± standard deviation. Samples were cured at five different temperatures (50 °C, 60 °C, 80 °C, 100 °C, and 120 °C), and measurements were taken at 0, 2, and 4 h. The data reveal a clear inverse relationship between the initial sealing pressure (at time = 0 h) and the curing temperature; samples cured at lower temperatures exhibited significantly higher initial pressures than those cured at higher temperatures. Specifically, the initial pressure was highest for the 50 °C sample (approximately 49 kPa) and lowest for the 120 °C sample (approximately 3 kPa).

The aging behavior over the 4 h test period varied significantly with curing temperature. Samples cured at 50 °C and 60 °C showed relatively slow degradation, maintaining a substantial portion of their initial pressure (declining to approx. 40 kPa and 35 kPa, respectively). In contrast, the sample cured at 80 °C, which started at a moderate pressure (approx. 31 kPa), exhibited a pronounced and relatively linear decrease, dropping to approximately 6 kPa after 4 h. Samples cured at 100 °C and 120 °C displayed poor initial sealing performance (approx. 6 kPa and 3 kPa, respectively). While the 100 °C sample showed minimal further pressure loss, the 120 °C sample’s sealing capability rapidly diminished to essentially zero within the 4 h timeframe. These results suggest that while lower curing temperatures yield higher initial sealing pressures, stability over time can vary, with 80 °C showing significant degradation. Conversely, curing at 100 °C or 120 °C results in intrinsically weak initial sealing.

Overall, this study highlights the critical role of PDMS curing conditions when using low-viscosity oils. Unlike previous studies [15,18,19,20] that primarily relied on high-viscosity silicone oils (e.g., 5000–10,000 cSt), where the effects of curing conditions were less significant, our results demonstrate that with low-viscosity oils (e.g., 120 cSt), the curing temperature of PDMS significantly influences interlayer adhesion. Lower curing temperatures enhance adhesion by increasing the number of tethered or free polymer chains on PDMS surfaces, which amplify van der Waals forces. This interplay between curing conditions and lubricant properties provides a simpler and more effective approach for achieving reliable sealing and controlled slipping performance in PDMS SlipChips.

### 3.2. Tensile Testing of PDMS Specimen Cured at Different Temperatures

**Figure 4:**
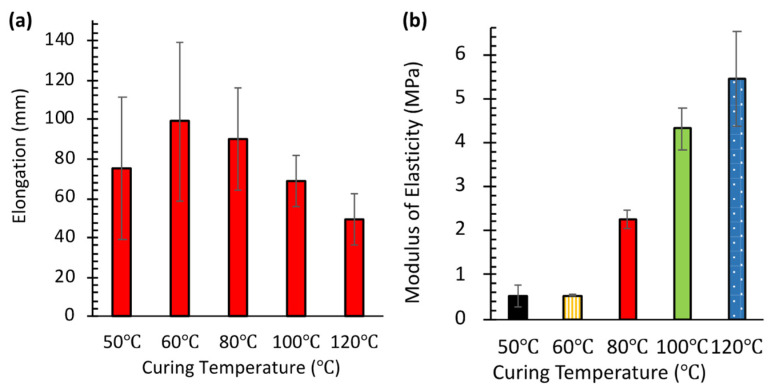
Effect of curing temperature on the mechanical properties of PDMS. (**a**) Elongation at break of PDMS samples cured at 50, 60, 80, 100, and 120 °C. (**b**) Modulus of elasticity of PDMS samples cured at 50, 60, 80, 100, and 120 °C.

Figure 4a presents the results of tensile testing performed in triplicate (*N* = 3) for each temperature condition. PDMS specimens cured at higher temperatures were more difficult to stretch and break, indicating increased rigidity. This rigidity correlates directly with higher cross-linking density. Conversely, specimens cured at lower temperatures (50–60 °C) exhibited greater elongation before failure, reflecting reduced cross-linking. This observation aligns with the higher burst pressures seen in Figure 3a. It suggests that the increased availability of free polymer chains on surfaces cured at lower temperatures enhances interlayer van der Waals adhesion.

Maximum elongation (99 ± 4 mm, mean ± SD, *N* = 3) was observed at 60 °C. This represents an optimal balance between cross-linking density and chain mobility, yielding both strength and flexibility. Interestingly, specimens cured at 50 °C showed slightly less elongation (75 ± 3 mm, mean ± SD, *N* = 3) compared to those at 60 °C. This suggests that very low curing temperatures might result in insufficient cross-linking, creating a more fragile network structure. Notably, these same 50 °C samples exhibited the highest burst pressure in pneumatic testing (Figure 3a).

Figure 4b quantifies the increase in stiffness using the modulus of elasticity, measured at 40% strain, which reflects the degree of cross-linking. The modulus remained low for specimens cured at 50 °C (0.51 ± 0.03 MPa, mean ± SD, *N* = 3) and 60 °C (0.53 ± 0.04 MPa, mean ± SD, *N* = 3). However, it increased sharply to 2.25 ± 0.12 MPa at 80 °C, and further rose to 4.31 ± 0.18 MPa at 100 °C and 5.45 ± 0.21 MPa at 120 °C. This progressive rise in modulus with curing temperature is consistent with previous findings by Konku-Asase et al. [21]. Although our values at 100 °C and 120 °C were slightly higher than their reported 3.7 MPa, the overall trend confirms that higher curing temperatures yield stiffer PDMS due to the increased in cross-linking density.

This observed increase in stiffness inversely correlates with the burst pressure results shown in Figure 3a. Together, these findings reveal a critical trade-off in SlipChip design. Lower-temperature curing enhances sealing properties (higher burst pressure) by producing softer PDMS with more free polymer chains available for adhesion. In contrast, higher-temperature curing improves structural integrity by creating stiffer materials but compromises interlayer adhesion because fewer free chains are available. Consequently, optimizing the curing temperature is essential for balancing mechanical strength and sealing performance in PDMS SlipChips.

### 3.3. Effects of Viscosity of Silicone Oils on Adhesion Between SlipChip Layers

Using the optimized PDMS curing protocol (top layer at 80 °C and bottom layer at 60 °C), we evaluated leakage performance using two different viscosity silicone oils. Figure 3b,c illustrates the leakage test results using an iodine solution. For devices coated with 50 cSt silicone oil, no leakage was observed when 200 µL of solution was injected over 10 s (equivalent to 20 µL/s flow rate), regardless of the spin coating speed (1000–2000 rpm). In contrast, devices coated with 100 cSt silicone oil showed different behavior depending on the spin coating speed. At lower speeds (1000 rpm and 1500 rpm), leakage occurred before reaching the target flow rate of 20 µL/s. However, at higher spin speeds (1800 rpm and 2000 rpm), the devices maintained proper sealing even at this flow rate.

This difference in sealing performance can be attributed to the nonuniform deposition of the higher viscosity 100 cSt silicone oil at lower spin speeds. Its higher viscosity causes the oil to form smaller droplets during spin coating, which later coalesce into larger bubbles [38]. These bubbles accumulate unevenly on the bottom PDMS layer, trapping air and increasing the separation between PDMS layers. This greater separation reduces van der Waals forces between layers, weakening adhesion and leading to leakage under high flow rates.

**Figure 5:**
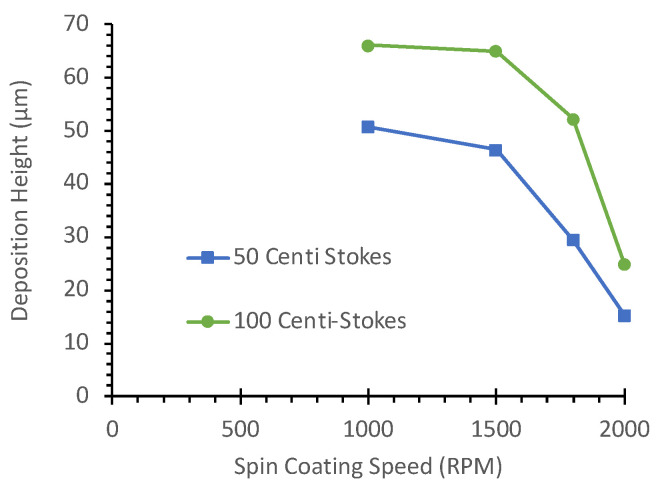
Deposition height of silicone oil on a bottom PDMS layer (cured at 60 °C) at different spin coating speeds.

Furthermore, as shown in Figure 5, the average deposition height of the oil gradually decreases with an increase in spin-coating speed. Average deposition heights for 50 cSt and 100 cSt silicone oils were measured at 1000 rpm, 1500 rpm, 1800 rpm, and 2000 rpm. At 1000 rpm and 1500 rpm, the average deposition height for 100 cSt silicone oil exceeded 60 µm in thickness, in contrast to the thinner coat for 50 cSt oil. Although these measurements were taken before assembling the SlipChip layers, they provide insights into the initial oil distribution patterns. The significant difference in deposition heights between 50 cSt and 100 cSt oils, particularly at lower spin speeds, helps explain the observed variations in sealing performance. The thicker oil layer formed by 100 cSt oil at low rpm likely contributes to the formation of larger bubbles and subsequent leakage issues when the layers are assembled.

### 3.4. Biocompatibility Test of Silicone Oils

**Figure 6:**
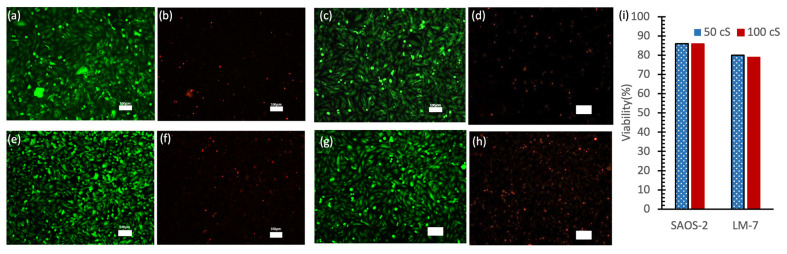
Live/dead cell assay for SAOS-2 and LM-7 cells with silicone oils in a 96-well plate. (**a**,**b**) SAOS-2 cells for 50 cSt oil. (**c**,**d**) SAOS-2 cells for 100 cSt oil. (**e**,**f**) LM-7 cells 50 cSt oil. (**g**,**h**) LM-7 cells 100 cSt oil. Scale bars (**a**–**h**) = 100 µm. (**i**) Cell viability.

Figure 6 presents the results of a live/dead cell analysis conducted after 48 h of cellular incubation in a 96-well plate. The fluorescence microscopy images show live cells stained with Calcein-AM (green fluorescence; Figure 6a,c,e,g) and dead cells stained with PI (red fluorescence; Figure 6b,d,f,h) for both SAOS-2 and LM-7 cells exposed to 50 cSt and 100 cSt silicone oils. The predominance of green fluorescence and minimal red fluorescence indicates high cell viability across all conditions. SAOS-2 cells (Figure 6a–d) particularly demonstrated uniform cell distribution and high viability under both oil viscosities.

Quantitative analysis (Figure 6i) revealed that SAOS-2 cells maintained approximately 86% viability in both 50 cSt and 100 cSt oils, while LM-7 cells showed slightly lower viability at approximately 80% and 79% in 50 cSt and 100 cSt oils, respectively. While a slight decrease in viability was noted with 100 cSt oil compared to 50 cSt oil, this difference was minimal and not statistically significant. Higher-viscosity oils may slightly limit oxygen and nutrient diffusion due to their denser structure [39,40].

These results are noteworthy because they demonstrate that low-viscosity silicone oil (50 cSt) can maintain high cell viability, a finding that is significant given previous studies showing that higher-viscosity silicone oils (e.g., 5000 cSt) are more cytotoxic and suppressive to cell growth [41,42]. This highlights the potential utility of 50 cSt silicone oil as a safer and less cytotoxic option for applications requiring prolonged cell culture.

### 3.5. Diffusion Mixing in SlipChip

**Figure 7:**
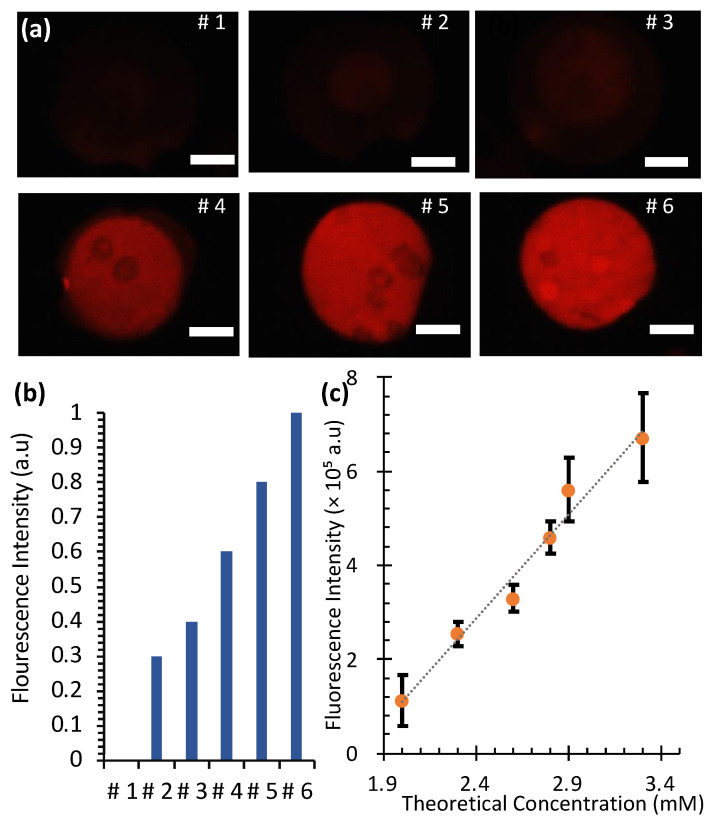
Fluorescence-based concentration gradient in SlipChip with 3.0 mm-diameter microwells. (**a**) Fluorescence images of microwells with various concentrations of Rhodamine 6G. Scale bars: 1 mm. (**b**) Normalized fluorescence intensity against each well number (**c**) Average fluorescence intensity against theoretical concentration.

Figure 7 illustrates the creation of a Rhodamine 6G concentration gradient in a SlipChip using ethanol as the solvent. Starting from a 4 mM stock solution, a single dilution was performed across six microwells with varying volumes, resulting in a concentration of 2.0 mM in the first well and 3.3 mM in the sixth well. Subsequent mixing in progressively smaller wells naturally created the gradient (Figure 7a).

To quantify the gradient, fluorescence intensities were measured for each microwell and normalized to facilitate comparison. The normalized intensity, denoted as $\bar{I}$, was calculated using the following formula:

$$\bar{I}=\frac{I-I_{min}}{I_{max}-I_{min}}$$

where I is the measured intensity, $I_{min}$ is the intensity of the lowest concentration (well 1), and $I_{max}$ is the intensity of the highest concentration (well 6). This normalization ensured that fluorescence intensity values ranged from 0 (well 1) to 1 (well 6), facilitating a direct comparison between experimental and theoretical values.

This normalization ensured that the intensity in well 1 was set to 0 ($\bar{I}=0$) and in well 6 it was set to 1 ($\bar{I}=1$), providing a standardized scale for comparing experimental and theoretical values. The relationship between fluorescence intensity (*I*) and Rhodamine 6G concentration (*C*) was found to be linear and is expressed as follows: *I* = 4.4 × 10^5^*C* − 7.8 × 10^5^. Here, *C* represents the Rhodamine 6G concentration in mM. The coefficient of determination (*R*^2^ = 0.98) indicates a strong correlation between fluorescence intensity and concentration.

The normalized fluorescence intensities, plotted in Figure 7b, showed a gradual increase from well 1 to well 6, consistent with the theoretical calculations. Statistical analysis (Figure 7c) confirmed a strong linear correlation between fluorescence intensity and theoretical concentration. These results validate that the SlipChip can generate reproducible concentration gradients, making it a reliable tool for biochemical assays requiring controlled diffusion and mixing.

### 3.6. SAOS-2 Cell Culture in PDMS SlipChip

**Figure 8:**
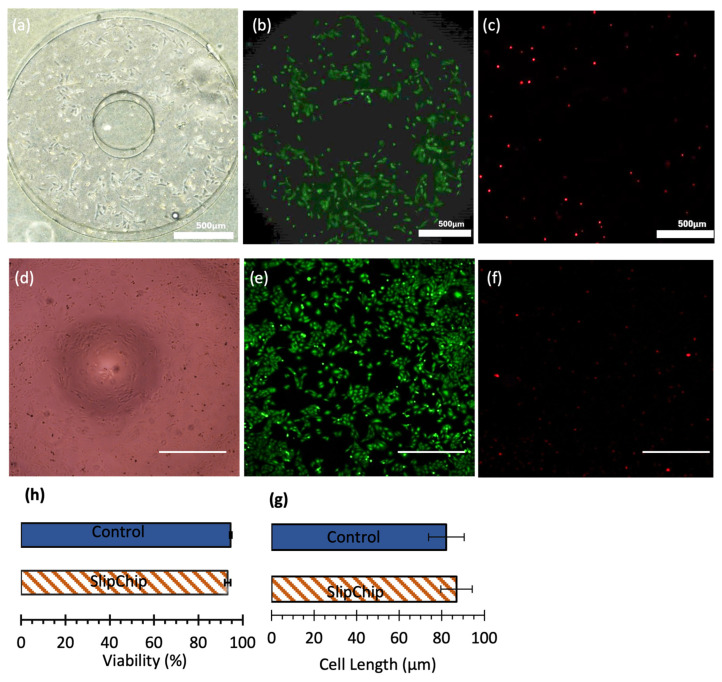
Comparison of cell culture in SlipChip and 96-well plate. (**a**–**c**) Bright-field, live (Calcein-AM-stained), and dead (PI-stained) cell images of SAOS-2 cells cultured in the SlipChip. Scale bar: 500 µm. (**d**–**f**) Bright-field, live (Calcein-AM-stained), and dead (PI-stained) cell images of SAOS-2 cells cultured in 96-well plates. Scale bars: 500 µm. (**g**) Average SAOS-2 cell length in the SlipChip (mean ± STD: 86.9 ± 7.3 µm) and 96-well plate (mean ± STD: 82.1 ± 8.4 µm). (**h**) Live and dead cell assay results showing comparable cell viability in both systems.

SAOS-2 cells were cultured in a PDMS SlipChip and a standard 96-well plate for a comparative study of cell health, morphology, and proliferation. Figure 8 illustrates the results of cell culture in both systems. Bright-field images (Figure 8a,d) show that SAOS-2 cells exhibited typical epithelial-like morphology in both systems, indicating good cell health. Calcein-AM staining (Figure 8b,e) confirms that cells were adequately elongated and spread, supporting their viability. The percentage of live cells after 24 h was 93.2% ± 1.3% (*N* = 3) in the SlipChip and 94.6% ± 0.5% (*N* = 2) in the 96-well plate (Figure 8h), demonstrating that the SlipChip provides a comparable environment for maintaining cell health.

Cell length was analyzed as an indicator of cell culture quality. Shorter cell lengths and rounded shapes typically signify a stressed state, whereas elongated cells indicate a relaxed state and healthy proliferation. As shown in Figure 8g, the average cell length of SAOS-2 cells was 86.9 µm (*N* = 62) in the SlipChip and 82.1 µm (*N* = 17) in the 96-well plate, consistent with previous findings of 78 µm, further supporting the suitability of the SlipChip for cell culture [38].

Cell proliferation was evaluated based on cell density after 24 h. Cells were seeded at an initial density of 100 cells/µL in both systems. After 24 h, the cell density reached 550 cells/µL (*N* = 3) in the SlipChip and 898 cells/µL (*N* = 2) in the 96-well plate. The slightly lower density observed in the SlipChip is likely due to medium evaporation caused by the porous nature of PDMS. Overall, these results demonstrate that PDMS is a biocompatible substrate for cell culture and that the three-dimensional encapsulated environment provided by the SlipChip is suitable for high-throughput biochemical analysis.

### 3.7. Comparative Analysis of SlipChip Performance

Table 1 provides a comprehensive comparison of different SlipChip systems across critical parameters including materials, lubricants, sealing methods, and biological applications. Our PDMS SlipChips with low-viscosity silicone oil (50 cSt) achieve performance comparable to high-viscosity alternatives while offering significant fabrication advantages. Chang et al.’s configuration using 10,000 cSt silicone fluid showed marginally higher cell viability (>98%) [15], but our direct spin-coating approach substantially simplifies manufacturing compared to their multi-step procedure, while maintaining similar culture durations and sealing performance.

The high cell viability in Chang et al.’s system using high-viscosity silicone fluid [15] may be attributed to their multi-step coating process, which likely creates an exceptionally thin lubricant layer that minimizes direct cell exposure while maintaining effective sealing. The direct application of high-viscosity oils would typically increase microchannel blockage rates. The comparable cell viability between mammalian systems confirms that low-viscosity oils effectively support cell culture without the blockage issues of higher-viscosity lubricants, providing a more accessible approach for general laboratory use.

The bacterial applications reported by Li et al. and Shen et al. demonstrate that for short-duration assays (30–60 min), both PDMS and glass SlipChips perform effectively, regardless of material choice [29,31]. While mammalian cell culture applications span multiple days—where material properties and gas permeability become critical factors—bacterial assays require significantly shorter timeframes, making the choice between glass and PDMS less consequential for these applications. This material flexibility is evidenced by successful bacterial studies using both PDMS with silicone oil (Li et al.) and glass with fluorinated oil (Shen et al.). The comparable performance across these different material configurations for short-term bacterial applications further validates SlipChip as a versatile platform adaptable to various biomedical applications with different temporal requirements.

## 4. Conclusions

This study addressed the limitations of high-viscosity silicone oils in PDMS SlipChips, such as channel blockages and impaired performance, by investigating low-viscosity alternatives and optimizing curing conditions. We demonstrated that curing the bottom layer at 60 °C and the top layer at 80 °C effectively balances adhesion and stiffness, ensuring reliable sealing and smooth slipping. Low-viscosity silicone oil (50 cSt) emerged as the optimal lubricant, resolving channel blockage issues while maintaining smooth slipping, effective sealing, and minimal cytotoxicity.

Biocompatibility testing further confirmed that 50 cSt oil supports osteosarcoma cell viability, comparable to traditional multiwell plates, making it suitable for biological assays and cell culture applications. This study underscores the significance of low-viscosity lubricants in conjunction with optimized curing conditions to overcome performance trade-offs, offering practical insights for developing biocompatible microfluidic systems. These findings on optimized sealing, slipping, and biocompatibility demonstrate the potential of PDMS-based SlipChips lubricated with low-viscosity oil for reliable mammalian cell studies, paving the way for applications such as drug discovery and protein detection assays.

## Figures and Tables

**Table 1:** Effects of the viscosity of silicone oil on the sealing performances and biocompatibility.

Material	Lubricant	Sealing Procedure	Cell Type	Culture/Assay Period	Cell Viability/Cytotoxicity	Reference
PDMS	50 cSt silicone oil	Direct spin-coat	Mammalian (SAOS-2)	24 h	>93% (live/dead assay)	This study
PDMS	10,000 cSt silicone fluid	Spin-coat, transfer, assemble	Mammalian (A549, MRC-5, ES-D3)	4–10 days	>98% (live/dead assay)	Chang et al., 2015 [15]
PDMS	Silicone oil	Spin-coat, transfer, assemble	Bacteria (*E. coli* O157:H7, *S. aureus*, *S. typhimurium*, *L. monocytogenes*)	30–60 min	Not specified (biosensing only)	Li et al., 2024, [31]
Glass	Fluorinated oil (FC-40)	Direct capillary fill	Bacteria (*E. coli* RP437, RP1616)	30–60 min	Not specified (recovered cells were culturable)	Shen et al., 2014 [29]

## Data Availability

The data presented in this study are available upon reasonable request from the corresponding author.
